# Dysregulated Expression of Glycolipids in Tumor Cells: From Negative Modulator of Anti-tumor Immunity to Promising Targets for Developing Therapeutic Agents

**DOI:** 10.3389/fonc.2015.00300

**Published:** 2016-01-07

**Authors:** Jose Luis Daniotti, Ricardo D. Lardone, Aldo A. Vilcaes

**Affiliations:** ^1^Centro de Investigaciones en Química Biológica de Córdoba (CIQUIBIC, UNC-CONICET), Departamento de Química Biológica, Facultad de Ciencias Químicas, Universidad Nacional de Córdoba, Córdoba, Argentina; ^2^Dirks/Dougherty Laboratory for Cancer Research, Department of Translational Immunology, John Wayne Cancer Institute at Providence Saint John’s Health Center, Santa Monica, CA, USA

**Keywords:** glycolipids, gangliosides, cancer, antibodies, immunotherapy, immunotoxin

## Abstract

Glycolipids are complex molecules consisting of a ceramide lipid moiety linked to a glycan chain of variable length and structure. Among these are found the gangliosides, which are sialylated glycolipids ubiquitously distributed on the outer layer of vertebrate plasma membranes. Changes in the expression of certain species of gangliosides have been described to occur during cell proliferation, differentiation, and ontogenesis. However, the aberrant and elevated expression of gangliosides has been also observed in different types of cancer cells, thereby promoting tumor survival. Moreover, gangliosides are actively released from the membrane of tumor cells, having a strong impact on impairing anti-tumor immunity. Beyond the undesirable effects of gangliosides in cancer cells, a substantial number of cancer immunotherapies have been developed in recent years that have used gangliosides as the main target. This has resulted in successful immune cell- or antibody-responses against glycolipids, with promising results having been obtained in clinical trials. In this review, we provide a general overview on the metabolism of glycolipids, both in normal and tumor cells, as well as examining glycolipid-mediated immune modulation and the main successes achieved in immunotherapies using gangliosides as molecular targets.

## Introduction

Differentially expressed tumor-associated carbohydrates represent a general phenomenon observed in many types of cancer cells. Carbohydrates covalently attached to glycolipids are not the exception. Neosynthesized glycolipids observed in oncogenic processes show antigen specificity and, therefore, they are attractive candidates for the design of cancer vaccines. The poor immunogenicity, low-affinity immunoglobulin responses, and immunotolerance associated with glycolipids have been overcome with the advent of new technologies and combinatorial immunotherapies. Nevertheless, these remarkable advances are being counteracted for striking effects on anti-tumor immunity exerted by particular molecular species of tumor-secreted glycolipids and, hence, strategies involving the use of glycolipid synthesis inhibitors are being considered.

## Glycolipid Metabolism and Functions

**Figure 1:**
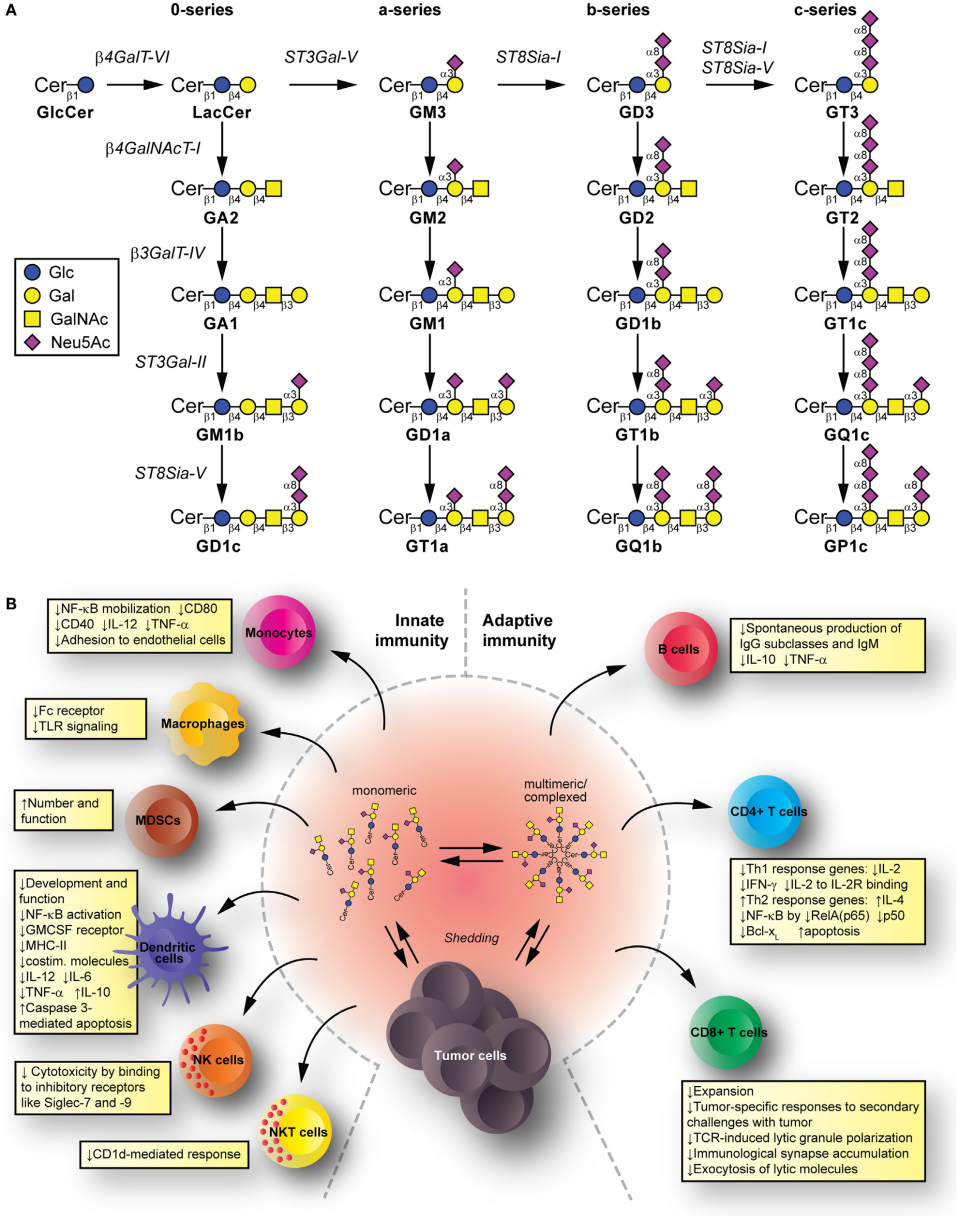
**Synthesis and immunomodulatory effect of gangliosides**. **(A)** Pathway for ganglioside biosynthesis representing the stepwise addition of monosaccharides to ceramide, and the resulting structures. β4GalT-VI, UDP-Gal:glucosylceramide galactosyltransferase; ST3Gal-V, CMP-NeuAc:lactosylceramide sialyltransferase; ST8Sia-I, CMP-NeuAc:GM3 sialyltransferase, and CMP-NeuAc:GD3 sialyltransferase; β4GalNAcT-I, UDP-GalNAc:lactosylceramide/GM3/GD3/GT3 *N*-acetylgalactosaminyl transferase; β3GalT-IV, UDP-Gal:GA2/GM2/GD2/GT2 galactosyltransferase; ST3Gal-II, CMP-NeuAc:GA1/GM1/GD1b/GT1c sialyltransferase; ST8Sia-V, CMP-NeuAc:GM1b/GD1a/GT1b/GQ1c sialyltransferase, and CMP-NeuAc:GD3 sialyltransferase. Cer, ceramide; Glc, glucose; Gal, galactose; GalNAc, *N*-acetylgalactosamine; Neu5Ac, *N*-acetylneuraminic acid (sialic acid). **(B)** Mechanisms for glycolipid-mediated immune modulation. Tumors shed gangliosides to extracellular milieu, where they are in dynamic equilibrium between monomeric, multimeric, and larger, hetero-complexed forms. From these various states, they have the potential to transfer to different immune cells, modify their membrane composition, and induce modifications that modulate innate and adaptive immunity. The summarized changes can thus favor tumor escape.

Glycolipids are molecules containing one or more carbohydrate residues linked to a hydrophobic lipid moiety via a β-glycosidic linkage. Those containing either a sphingoid or a ceramide as the hydrophobic lipid moiety are referred to as glycosphingolipids (GSLs). A particular subclass of glycolipids is the gangliosides, which are sialylated GSLs mainly expressed in the outer layer of the plasma membrane of essentially all vertebrate cells. The biosynthesis of gangliosides starts with the synthesis of ceramide at the cytoplasmic leaflet of the endoplasmic reticulum membrane, where the pyridoxal 5′-phosphate-dependent serine palmitoyltransferase catalyzes the condensation of palmitoyl- or stearoyl-Coenzyme A with l-serine to give 3-ketosphinganine, which is reduced to d-erythro-sphinganine by 3-ketosphinganine reductase in a NADPH-dependent reaction. d-erythro-sphinganine is further acylated to generate different dihydroceramides by a family of ceramide synthases. Next, dihydroceramide is unsaturated at the 4,5 position by DES1 desaturase to make ceramide. The *de novo* synthesized ceramide is then transported from the endoplasmic reticulum to the *trans* Golgi, at least in part in a protein-dependent manner by the transport protein CERamide Transport (CERT), where it is catalytically converted to glucosylceramide (GlcCer) by the action of UDP-Glc:ceramide glucosyltransferase. Most GlcCer may subsequently be transported by the four-phosphate adaptor protein 2 (a glycolipid-transport protein carrying a PI4P-binding domain) either to the endoplasmic reticulum or to distal Golgi compartments, where it translocates to the lumen. β4GalT-VI converts GlcCer to lactosylceramide (LacCer) and further carbohydrate residues, including negatively charged sialic acid, are transferred in a stepwise manner to the growing glycan chains (Figure 1A). Sialylated derivatives from LacCer are produced by the action of ST3Gal-V, ST8Sia-I, and ST8Sia-I/ST8Sia-V, which specifically catalyze the formation of the gangliosides GM3, GD3, and GT3, respectively. LacCer, GM3, GD3, and GT3 serve as precursors for more complex gangliosides of the 0-, a-, b-, or c-series by sequential glycosylations catalyzed by β4GalNAcT-I, β3GalT-IV, ST3Gal-II, and ST8Sia-V. After synthesis at the Golgi complex, gangliosides are mainly delivered by vesicular transport to the plasma membrane, where they can undergo endocytosis. In addition to the bulk ganglioside synthesis at the Golgi complex level, ganglioside formation by plasma membrane-associated glycosyltransferases has been recently also reported (1–4). See Ref. (5–9) for an extensive review on ganglioside biosynthesis and molecular transport pathways.

The catabolism of gangliosides takes place mainly at the lysosomes, although degradation of gangliosides can also occur at the cell surface by the action of the sialidase Neu3, β-galactosidase, and β-glucosidase (10–14). At the lysosomal level, gangliosides are sequentially degraded by the action of glycosidases that sequentially cleave off the monosaccharide units from the non-reducing end of the ganglioside glycan chains. Adequate lysosomal ganglioside catabolism requires the presence of an appropriate pH, suitable glycosidases, and lipid-transfer proteins for the degradation of simple gangliosides, which extracts the membrane-bound glycolipids and presents them to the soluble acid hydrolase [see Ref. (15–17) for an extensive review on pathways of ganglioside catabolism].

Ganglioside expression changes with cell growth, differentiation, viral transformation, oncogenesis, and ontogenesis (18–21). Gangliosides, originally identified as structural components of biomembranes, were later acknowledged as key lipids implicated in a range of cellular processes. Thus, gangliosides are involved in many physiological processes, including growth, differentiation, migration, and apoptosis through modulating both cell signaling processes and cell-to-cell and cell-to-matrix interactions (22–28). Moreover, gangliosides have been associated with a wide range of pathological processes, being receptors for viruses, toxins, and autoantibodies associated with clinically identifiable acute and chronic neuropathy syndromes. In addition, inherited defects in the biosynthesis or degradation of gangliosides have also been described, which causes a group of diseases mainly associated with severe neurodegenerative disorders (29–34).

Although the plasma membrane is the major cellular reservoir of gangliosides, it is not the final destination for these molecules. Thus, in addition to cell internalization, sorting to lysosomes or plasma membrane recycling, gangliosides can be actively shed from the membrane of one cell and taken up by other cells by insertion of their lipid anchors into the cell membrane. Although the shedding and uptake of gangliosides are a physiological process observed in many types of cells, increased levels have been detected in various tumors, such as melanomas, renal carcinoma, astrocytomas, and glioblastomas (35–39). Many reports have described that shedding of gangliosides helps to suppress the immune response, probably by modifying membrane composition of immune cells and, hence, inhibiting their function and allowing tumor escape. This topic is further reviewed below.

## Glycolipid-Mediated Immune Modulation

Tumors have a complex interrelation with the immune system. The immunoediting paradigm (40) describes how pressure exerted by immune cells recognizing and eliminating tumor cells results in the selection for poorly recognized malignant cells. Tumors can further disrupt this apparent equilibrium by inducing an immunosuppressive environment that allows tumor cells escape (41). Gangliosides shed from tumors and reaching immune cells have potential to contribute mechanisms suppressing the immune system. *Innate immunity* cell populations, such as monocytes, macrophages, and dendritic cells (DC), kill tumor cells directly, release inflammatory mediators to recruit and differentiate adaptive immune cells, and present tumor antigens to T cells (42–44). Natural killer (NK) cells kill tumors directly depending on the balance between inhibitory and activating signals from invariant receptors (45). Natural killer T (NKT) cells can target lipid and glycolipid antigens in the context of (MHC-I resembling) CD1 molecules and mediate anti-tumor effects (46). Among the *adaptive immunity* cell populations, we find B cells (producing anti-tumor antibodies targeting cancer cells for killing by effector cells, and presenting antigen to T cells), as well as CD4+ T cells (helping with antibody production and cell-mediated immune responses) and CD8+ T cells [effector cells for tumor killing (47)]. The immunomodulatory effects by gangliosides take place to the level of both, the innate and the adaptive immunity (Figure 1B).

### Mechanisms Suppressing Innate Immunity

Human brain gangliosides impede up-regulation of the costimulatory molecule CD80 (without affecting expression of I-CAM-1, LFA-3, HLA-DR, and CD86) on monocytes (48). Similarly, exposing monocytes to GD1a also inhibits CD80 up-regulation, decreases CD40 levels, and reduces LPS-stimulated interleukin (IL)-12 and TNF-α (49) by impeding NF-κB mobilization (50). In addition, pre-incubation of monocytes with certain gangliosides can impair Fc receptor expression (by GM2 and GM3), IL-1 production (by GM1 and GD3) (51), and TLR signaling (52). Moreover, GM3 reduces the monocyte adhesion to endothelial cells (53). Importantly, tumor-derived gangliosides can increase number and function of myeloid-derived suppressor cells to favor immune escape (54).

Gangliosides, such as GM2 (55), GD1a (56), GM3, and GD3 (57), can affect *in vitro* development and function of monocyte-derived DC. Expression of MHC class II, costimulatory molecules, and CD116 (GM-CSF receptor) on DC is reduced by GM2. Endocytic, chemotactic, and T cell proliferation-inducing activities are also targeted. Meanwhile, GD1a mediates a poor DC response to activating conditions by reducing costimulatory molecules, IL-12, TNF-α, and IL-6 production, and increasing IL-10 release (49, 56), presumably through NF-κB activation disruption. This impaired response of activated DC is also observed with GM3 and GD3 (57), which also induce caspase 3-mediated apoptosis (57–59). NK cell cytotoxicity against tumor cells is reduced by tumor gangliosides binding to inhibitory receptors such as Siglec-7 and -9 (60–62). Finally, gangliosides can also interfere with NKT cells activation, such as GD3 in ovarian cancer (63) and GM2 in lymphoma (64), often acting as inhibitory ligands for the CD1d-mediated NKT cell response.

### Mechanisms Suppressing Adaptive Immunity

GM2 and GM3 gangliosides added *in vitro* to B cells inhibit spontaneous production of IgG subclasses and IgM, with no effect on IgA subclasses (65). At least for GM2, the mechanism involves reduction of endogenous IL-10 and TNF-α production (66), while the presence of TNF-α can counteract these inhibitions. In addition, certain complex gangliosides can affect IL-6 and IL-10 production on CD4+ T cells (GD1b) and monocytes (GT1b) (67, 68), also leading to further reduced IgG, IgM, and IgA antibody production in co-cultures with B cells. Remarkably, GQ1b and GD1a can abrogate the effects of GD1b and GT1b to enhance Ig production by human peripheral blood mononuclear cells (69, 70). It is noteworthy to mention that no effects have been described for gangliosides on antigen presentation by B cells.

Regarding T cells, gangliosides can have effects at both, central (71) and peripheral (72) T cell compartments. The induction of cytolytic anti-tumor immunity relies on type-1 T cell responses [conducted by interferon (IFN)-γ- and IL-2-producing T cells (73)], as opposed to type-2 T cell responses (defined by T cells producing IL-4, IL-6, and IL-10) leading to cytolytic activity suppression (74). Indeed, cancer patients produce increased type-2 cytokines (75). Several genes participate in T cell development, maturation, and proliferation, under transcriptional control of Rel/NF-κB (76). Renal cell carcinoma (RCC)-derived gangliosides reduce IL-2 and IFN-γ expression (77) and increase apoptosis in T cells through NF-κB inhibition by reducing RelA(p65), p50, and antiapoptotic protein Bcl-x_L_ (78). Tumor- or brain-derived gangliosides present during *in vitro* activation-induced settings (e.g., with anti-CD3 antibody) also associate with type-2 response shifts in CD4+ and CD8+ T cell populations by decreasing IFN-γ and often elevating IL-4 (79–81), along with increased apoptosis. Interestingly, *in vivo* findings show more apoptotic T cells in RCC patients having GM2 in their membranes but with negligible mRNA expression levels for GM2 synthase (82). This ectopic presence of GM2 could derive from the tumor and participate in apoptosis mediated by diverse mechanisms (82–86). Antigen-specific T cell activation is also interfered by the presence of gangliosides. These effects are mediated by IL-2 transcription blockade and phosphorylation inhibition of retinoblastoma protein in activated human T cells (81). Moreover, ganglioside mixtures can inhibit proliferation of IL-2-dependent T cell lines by a competitive inhibition for IL-2 binding to IL-2R, mediated by direct binding of gangliosides to a lectin-like site on IL-2 (87).

*In vivo* mice models indicate that cytotoxic CD8+ T cell populations are also affected by gangliosides exposure, in terms of expansion and tumor-specific responses to secondary challenges with tumor cells (88). In addition, gangliosides prevent TCR-induced lytic granule polarization, immunological synapse accumulation, and exocytosis, without interfering on lytic molecule expression or target cell recognition (89).

Despite all these detrimental effects, the bright side of ganglioside dysregulation in tumors is the opportunity to aim them as molecular targets. Next section presents current promising immunotherapy strategies based on this principle.

## Immunotherapies Using Gangliosides as Molecular Targets

Among the reported changes on lipid composition of tumor cell membranes, the remarkable modifications on the sialic acid-containing glycolipids during neoplastic transformations have received special attention. This spectrum also includes molecules such as lacto- and neolacto-series glycolipids and globosides (GSLs containing acetylated amino sugars and simple hexoses). Tumors, such as melanoma, small-cell lung cancer (SCLC), sarcoma, and neuroblastoma, express gangliosides GD3, GM2, and GD2 in higher levels than corresponding normal tissue (90–94). Moreover, ganglioside derivatives, including *N*-glycolyl GM3 (NeuGcGM3) and fucosyl-GM1, are also increased (94–97). Therefore, a substantial number of cancer immunotherapies have been using sialylated glycolipids as major targets. However, no glycolipid-containing cancer vaccines have produced substantial clinical improvements. Nevertheless, several reviews have recently covered in depth and from different perspectives a renewed interest in therapeutic applications of glycoconjugates. Due to the stronger support based on the recent development of new tools and techniques, along with advances in the glycobiology field and potential combination with other therapeutic approaches, the authors share a common denominator: the re-emergence of gangliosides as promising targets for developing cancer therapeutic agents (18, 41, 98–102). An example is GD3, highly expressed in tumor cells (>80% of melanomas) (90). Investigations focusing on this ganglioside as principal target were made in passive (103) and active (104) immunotherapy of melanoma cancer, with modest results (105). However, a recent strategy is evaluating a GD3-specific chimeric antigen receptor (CAR) to redirect T cell specificity to GD3 expressed on tumor cells surface (90) (Table 1). Recently, GD3 has been proposed as a suitable immunotherapy target for tumors developed in lymphangioleiomyomatosis (106). In this sense, the mouse monoclonal R24 antibody (IgG3) against ganglioside GD3 is a validated tumor targeting agent (107). Our laboratory demonstrated that the R24 antibody, after binding to GD3 at the cell surface, is rapidly internalized and accumulated in endosomal structures (108). We took advantage of this internalization feature for selectively delivering the toxin saporin (a ribosome-inactivating protein) to GD3-expressing human (SK-Mel-28) and mouse (B16) melanoma cells (109). This represented the first proof-of-concept of an original strategy in which a glycolipid emerges as a novel and attractive class of cell surface molecule for targeted drug delivery. Recently, this experimental strategy was also used for selective ablation of cell lines expressing 9-*O*-acetyl GD3 (110). Thus, ganglioside GD3 re-emerges as an attractive cell surface molecule for targeted delivery of cytotoxic agents such as saporin or, eventually, other drugs such as paclitaxel (Taxol) and doxorubicin (111, 112).

**Table 1:** **Current and promising immunotherapeutic strategies involving tumor-associated gangliosides**.

Ganglioside	Type of treatment	Description	Type of acquired immunity	Phase of clinical research	Type of human tumor	Reference
*N*-glycolyl-GM3	Anti-idiotype Ab (racotumomab)	Murine gamma-type anti-idiotype monoclonal antibody that specifically induces an antibody response to Neu5Gc-containing gangliosides, sulfatides, and other antigens expressed in tumors	Active	Phase III trial	Non-small-cell lung cancer	(102, 113, 114)
GD2	A chimeric Hu-murine antibody	Anti-GD2 Ab Ch14.18 + GM-CSF + IL-2 + isotretinoin	Passive	Phase I trial	High-risk neuroblastoma	(115)
		Anti-GD2 Ab (hu14.18K322A). Humanized anti-GD2 Ab with a single point mutation (K322A) that reduces complement-dependent lysis		Phase I trial	Refractory or recurrent neuroblastoma	(116)
	Immunocitokine chimeric hu14.18 Ab-IL2	Hu14.18-IL2 fusion protein consists of interleukin-2 molecularly linked to a humanized monoclonal antibody that recognizes the GD2		Phase II trial	Relapsed/refractory neuroblastoma	(117)
					metastatic melanoma	(118, 119)
	CAR	Natural killer-92 cells stably express a GD2-specific CAR, which carries a cell-binding domain derived from antibody ch14.18		Preclinical	–	(120)
		Cell line: Hu neuroblastoma				
	Anti-idiotype Ab (gangliomab)	Immunization of Balb/c mice with 14G2a (murine monoclonal antibody to GD2), and splenocytes were harvested to generate hybridoma cells. Clones were screened for mouse antibody binding to hu14.18	Active	Preclinical	–	(121)
		Cell line: Hu neuroblastoma				
	Inhibitor	Triptolide, a small molecule inhibitor, inhibits ST8-SiaI expression, GD2 biosynthesis, and cancer stem cells-associated properties	–	Preclinical	–	(122–124)
		Cell line: Hu breast cancer/human tissue				
GD3	Targeted delivery of cytotoxic agents	Secondary antibody coupled to Zap bounded to the mouse antibody to GD3 R24	–	Preclinical	–	(109)
		Cell line: Hu and mouse melanoma				
	Targeted delivery of cytotoxic agents	Secondary antibody coupled to Zap bounded to NG2 and a GD3A9-*O*-acetyl GD3 antibodies	–			(110)
		Cell line: Hu glioblastoma multiforme				
	CAR	Anti-GD3 tandem chimeric sFv-CD28/T-cell receptor zeta designer T cells. Second generation	Passive			(90)
		Cell line: human melanoma. Model animal: BALB/c nude mice				
GM2	Synthetic carbohydrate-based vaccines	Unimolecular pentavalent construct KLH conjugate (UPC-KLH, 2). Five prostate and breast cancer-associated carbohydrate antigens, globo-H, GM2, STn, TF, and Tn conjugated to the carrier protein KLH	Active	Preclinical	–	(125)
		Model animal: mice (C57BL/6J)				

*Ab, antibody; NG2, neuron-glia 2 (a transmembrane chondroitin sulfate proteoglycan); CAR, chimeric antigen receptors; Hu, human; KLH, keyhole limpet hemocyanin; Tn antigen, Thomsen-nouvelle antigen (GalNAcα1-*O*-Ser/Thr); STn, sialyl-Tn; TF, Thomsen–Friedenreich antigen; Zap, saporin*.

One of the strategies to generate an effective immune response against tumor-associated carbohydrate antigens (TACAs) involves the use of anti-idiotype antibodies as antigen surrogates. Although several investigations support the role of GM3 in suppression of cancer development and progression (99), overexpression of NeuGcGM3 has received special attention because it is minimally expressed on most normal human tissues. In the 1990s, it was described an anti-idiotypic murine monoclonal antibody (1E10) that reacts with a monoclonal IgM antibody (P3) binding to *N*-glycolyl-containing gangliosides. 1E10, commercially named racotumomab, mimics NeuGcGM3 ganglioside to induce a strong anti-metastatic effect in tumor-bearing mice (126). Racotumomab is being evaluated for a wide range of NeuGcGM3-expressing tumors such as melanoma, breast cancer, non-SCLC, and several pediatric tumors of neuroectodermal origin (113, 127–133). A Phase II/III multicenter double-blind clinical trial evaluated racotumomab vaccine effects in the overall survival in advanced non-SCLC patients (134). This study showed a significant clinical benefit for patients treated with the anti-idiotype vaccine. On the basis of these promising results, racotumomab was launched in 2013 in Cuba and Argentina as an intradermal injection for treating patients with advanced stage non-SCLC (113). It is worth mentioning that naturally occurring antibodies against TACAs have been detected in both, cancer patients and healthy donors (135, 136). In addition, TACA-specific antibodies were screened in pooled sera of thousands of healthy donors. Notably, a structure–immunogenicity relationship was observed (137). Thus, idiotypic vaccination could be an optimal way to activate immune response cascades involving the natural responses against these antigens. Recent results suggest the existence of antibodies against NeuGcGM3 with anti-tumor immune surveillance functions, reinforcing the importance of N-glycolylated gangliosides as anti-tumor targets (138, 139).

Other TACAs are GD2, ranking 12 out of 75 potential targets for cancer therapy by National Cancer Institute pilot program for the prioritization of the most important cancer antigens (140). Combination of anti-GD2 antibody (ch14.18) with IL-2 and GM-CSF significantly improves survival for high-risk neuroblastoma patients (141, 142). The aforementioned study reflects the need to combine cancer immunotherapeutic treatments with other interventions. In this sense, safe transfer of CAR-based immunotherapy into clinical practice represents a potential alternative to conventional treatment options for cancer patients. Similar to GD3, CAR technology associated with GD2 has already shown significant anti-tumor activity in neuroblastoma patients (143, 144).

As mentioned in the previous section, tumor cells shed gangliosides and populate their microenvironment with these and other biologically active GSLs. Recently, strong evidences indicate that gangliosides synthesized and released by tumor cells have critical proangiogenic activity *in vivo*, which is associated with enhanced tumor growth (145). These findings indicate that inhibition of human tumor ganglioside synthesis could be a novel therapeutic target for human cancer. At this respect, *N*-butyldeoxynojirimycin, an imino-sugar administered orally to inhibit GlcCer synthase, delayed tumor development of MEB4 murine cells (146). This could be further explored in human cancer as a therapeutic approach, aiming at intervene on GSL metabolism of tumor cells and modulate GSLs shedding, thus lessening the immunomodulatory effects of GSLs (39).

Glycoengineering is another promising therapeutic approach for cancer (147). Essentially, a spectrum of scientific disciplines, such as carbohydrate chemistry, chemical biology, and glycobiology, converge for creating improved or novel glycan products to control human health and disease (147). For instance, the first globo-H vaccine for clinical use was developed in 2001 (148). The cell-surface GSL globo-H is a member of a family of tumor-associated antigens highly expressed on several types of cancers. Then, an optimized vaccine against the globo-H containing five prostate and breast cancer-associated carbohydrate antigens including GM2 was reported (125). More recently, a globo-H vaccine with different carriers and adjuvants was developed to improve the immunogenicity and safety profile (149). Over the last few years, some studies demonstrated an efficient metabolic glycoengineering of GM3 on melanoma cells with monoclonal antibody-mediated selective killing of glycoengineered cancer cells. Basically, cells were metabolically labeled both *in vivo* and *in vitro* with *N*-phenylacetyl-d-mannosamine (ManNPhAc) and then selectively targeted and killed with a monoclonal antibody (2H3) recognizing both GM3NPhAc and ManNPhAc (150, 151).

## Concluding Remarks

Cells becoming cancerous develop profound metabolic changes that influence plasma membrane composition. The expression/overexpression of TACAs antigens such as gangliosides on cancer cell surface are involved in tumor evasion from the immune response. However, cancer therapy can exploit this undesirable expression as targets. Table 1 summarizes some of the current and promising cancer treatments aiming at gangliosides. Monoclonal antibodies clearly represent one of the most important strategies employed (152). Recent studies demonstrated how the levels of antibody-mediated inverse hormesis could differentially influence tumor growth (153). These findings may have important implications for cancer immunotherapy with antibodies, including those against glycolipids. Remarkable glycoengineering advances for the development of new and better anticancer antibodies, along with the design of novel and specific inhibitors of glycolipid synthesis or the precise delivery of cytotoxic agents, are renewing the applicability of gangliosides as targets for cancer therapy.

## Author Contributions

JD, RL, and AV contributed to the conception and design of the work. All authors wrote, edited, and reviewed the final manuscript version.

## Conflict of Interest Statement

The authors declare that the research was conducted in the absence of any commercial or financial relationships that could be construed as a potential conflict of interest.
